# MRP8/14 serum levels as a predictor of response to starting and stopping anti-TNF treatment in juvenile idiopathic arthritis

**DOI:** 10.1186/s13075-015-0723-1

**Published:** 2015-08-07

**Authors:** Janneke Anink, Lisette W. A. Van Suijlekom-Smit, Marieke H. Otten, Femke H. M. Prince, Marion A. J. van Rossum, Koert M. Dolman, Esther P. A. H. Hoppenreijs, Rebecca ten Cate, Simona Ursu, Lucy R. Wedderburn, Gerd Horneff, Michael Frosch, Thomas Vogl, Faekah Gohar, Dirk Foell, Johannes Roth, Dirk Holzinger

**Affiliations:** Department of Pediatrics/ Pediatric Rheumatology, Erasmus MC Sophia Children’s Hospital Rotterdam, Rotterdam, The Netherlands; Department of Pediatrics/ Pediatric Rheumatology Academic Medical Centre Emma Children’s Hospital and Reade location Jan van Breemen, Amsterdam, The Netherlands; Sint Lucas Andreas Hospital and Reade location Jan van Breemen, Amsterdam, The Netherlands; Sint Maartenskliniek and Radboud University Nijmegen Medical Centre, Nijmegen, The Netherlands; Leiden University Medical Centre, Leiden, The Netherlands; Infection, Immunity, Inflammation and Physiological Medicine Programme UCL Institute of Child Health, University College London, London, UK; Centre of Pediatric Rheumatology, Department of General Pediatrics, Asklepios Clinic Sankt Augustin, Sankt Augustin, Germany; German Pediatric Pain Centre, Children’s and Adolescents’ Hospital, Datteln, Germany; Institute of Immunology, University Hospital Muenster and Interdisciplinary Centre for Clinical Research IZKF, University Hospital Muenster, Muenster, Germany; Interdisciplinary Centre for Clinical Research IZKF, University Hospital Muenster, Muenster and Department of Pediatric Rheumatology and Immunology, University Children’s Hospital Muenster, Muenster, Germany

## Abstract

**Introduction:**

Approximately 30 % of juvenile idiopathic arthritis (JIA) patients fail to respond to anti-TNF treatment. When clinical remission is induced, some patients relapse after treatment has been stopped. We tested the predictive value of MRP8/14 serum levels to identify responders to treatment and relapse after discontinuation of therapy.

**Methods:**

Samples from 88 non-systemic JIA patients who started and 26 patients who discontinued TNF-blockers were analyzed. MRP8/14 serum levels were measured by in-house MRP8/14 ELISA and by Bühlmann Calprotectin ELISA at start of anti-TNF treatment, within 6 months after start and at discontinuation of etanercept in clinical remission. Patients were categorized into responders (ACRpedi ≥ 50 and/or inactive disease) and non-responders (ACRpedi < 50) within six months after start, response was evaluated by change in JADAS-10. Disease activity was assessed within six months after discontinuation.

**Results:**

Baseline MRP8/14 levels were higher in responders (median MRP8/14 of 1466 ng/ml (IQR 1045–3170)) compared to non-responders (median MRP8/14 of 812 (IQR 570–1178), p < 0.001). Levels decreased after start of treatment only in responders (p < 0.001). Change in JADAS-10 was correlated with baseline MRP8/14 levels (Spearman’s rho 0.361, p = 0.001). Patients who flared within 6 months after treatment discontinuation had higher MRP8/14 levels (p = 0.031, median 1025 ng/ml (IQR 588–1288)) compared to patients with stable remission (505 ng/ml (IQR 346–778)). Results were confirmed by Bühlmann ELISA with high reproducibility but different overall levels.

**Conclusion:**

High levels of baseline MRP8/14 are associated with good response to anti-TNF treatment, whereas elevated MRP8/14 levels at discontinuation of etanercept are associated with higher chance to flare.

**Electronic supplementary material:**

The online version of this article (doi:10.1186/s13075-015-0723-1) contains supplementary material, which is available to authorized users.

## Introduction

Addition of biologic agents for treatment of juvenile idiopathic arthritis (JIA) has brought the treatment goal of inactive disease into reach even for JIA patients not responding to conventional disease-modifying anti-rheumatic drugs (DMARDs). However, 30–40 % of patients treated with biologic agents do not achieve this treatment goal for unknown reasons [1–3]. Several clinical parameters have been found to be associated with response to etanercept, a TNF-alpha inhibitor and the first biologic agent to be approved for the treatment of JIA [4, 5]. These include patient characteristics, such as age and gender, and disease characteristics, such as number of active joints, extent of disability and disease duration. However, as none of these factors are perfectly able to distinguish between responders and non-responders, these clinical characteristics in themselves are not sufficient to guide treatment decisions. A more tailored approach to drug choice, based upon use of validated biomarkers in combination with clinical parameters, could facilitate early remission induction for more children. Measurement of serum inflammatory proteins before starting treatment with a biologic agent may be valuable to separate children with a high chance of good response from poor responders or non-responders. In addition, biomarkers could be of help in identifying patients in clinical remission who can successfully discontinue treatment.

The myeloid related protein (MRP) complex 8/14 (S100A8/9, also known as calprotectin) is released from activated monocytes and phagocytes. MRP8/14 is a ligand to toll-like receptor 4 (TLR-4), has a pro-inflammatory effect on phagocytes and endothelial cells [6] and is an important factor in mediating osteoclastic bone destruction in experimental arthritis [7]. MRP8/14 serum levels correlate with disease activity in JIA patients [8], can be used to identify subclinical disease activity, and are associated with flares in JIA patients in clinical remission on methotrexate (MTX) [9, 10]. In addition, this biomarker correlates closely to response to treatment in patients with systemic JIA [11] and is able to predict good response to MTX in a subset of patients with non-systemic JIA [12].

Whether MRP8/14 is also associated with response to TNF-alpha inhibitors in patients with non-systemic JIA, or can predict flares after discontinuation of etanercept after successful treatment when clinical remission is achieved, is unknown. Therefore, in the present study we prospectively evaluated the relationship between the clinical course of JIA after anti-TNF treatment and after discontinuation of etanercept and serum levels of MRP8/14.

## Methods

### Study population

Serum samples were included from patients with non-systemic JIA who were biologic-agent-naïve, starting either etanercept or adalimumab, and included in the Dutch Arthritis and Biologicals in Children (ABC) Register (n = 68), German Registry for Biologics in Pediatric Rheumatology (BIKER) (n = 12) or Childhood Arthritis Response to Medication Study (CHARMS) from the United Kingdom (n = 8). Additionally samples from 26 patients at discontinuation of etanercept in remission were collected ((ABC register (n = 8), BIKER register (n = 18)). The list of participating hospitals can be found in the Acknowledgements section. Patients fulfilled the International League of Associations for Rheumatology criteria for JIA [13]. Patients diagnosed with systemic JIA have been described elsewhere [11].

The ABC register is a multicenter prospective observational study that aimed to include all JIA patients in the Netherlands who initiated biologic agents. The study protocol was approved by the Medical Ethics Committee at Erasmus MC Rotterdam and by all participating hospitals [3]. The German BIKER register was founded with the same objective, after approval by the ethics committee of the University Halle [1]. The CHARMS study (approved by the Institute of Child Health/Great Ormond Street NHS Trust Ethics Committee) included JIA patients at the start of treatment with new disease-modifying medication for active arthritis [12]. For all studies, written informed consent was obtained. In all three studies patient and disease characteristics were recorded at the start of biologic treatment. Changes in disease activity, medication use and adverse events were followed prospectively. These included the JIA core set variables: physician’s global assessment (PGA) of disease activity on a visual analog scale (VAS) (range 0–10 cm, 0 best score), childhood health assessment questionnaire (CHAQ) (range 0–3, 0 best score), the global assessment of wellbeing VAS completed by patients/parents (range 0–10 cm, 0 best score, number of joints with active arthritis and joints with limited motion and erythrocyte sedimentation rate (ESR)). Additionally pain was assessed using a VAS completed by parents/patients. Anti-TNF treatment was prescribed according to standard dosing for pediatric JIA patients.

### Response to treatment, inactive disease and flare

The effect of treatment was assessed using the ACR pediatric response criteria [14]. A modified definition for inactive disease was used and defined as no active arthritis, no systemic features, no uveitis, normal ESR (≤20 mm/h), and physician’s global assessment of disease activity indicating no disease activity (defined as a score ≤1.0 cm) [15]. The modified definition includes PGA with an increased threshold, because in daily practice we experience a fear of physicians to set the disease activity at zero, as while the disease is inactive, patients are not cured and still require medication. Patients were divided into responders who achieved at least an ACRpedi50 response (subdivided into ACRpedi50, ACRpedi70 or inactive disease) and non-responders (patients with no response or an ACRpedi30 response) within 6 months after start of treatment (median time of follow-up evaluation was 3.2 months (IQR 2.6 − 5.0). Additionally, response to treatment was evaluated using change on the continuous juvenile arthritis disease activity score (JADAS)-10 score, a composite score based on four of the disease activity variables (PGA, VAS wellbeing (parent/patient), active joint count and ESR). JADAS-10 was assessed at the start and within 6 months after the start of treatment [16].

Patients who discontinued treatment were all in remission on medication (defined as a period of ≥6 months of continuous inactive disease, using the modified Wallace criteria as described above) [17]. For evaluation of the association between MRP 8/14 levels at discontinuation and flaring after discontinuation, we defined flare as having at least three of the following: a physician or patient VAS ≥20 mm, ≥1 active joints, any worsening on the CHAQ and ≥30 % worsening of ESR and limited joints [18, 19]. Patients who had flares no longer fulfilled the modified Wallace criteria. Patients who did not have flares still met these criteria.

### Determination of MRP8/14 serum levels

Serum levels of MRP8/14 complexes were determined by ELISA. For comparison with earlier studies, internal control sera were used as a reference in all ELISA studies. MRP8/14 serum levels in healthy pediatric controls have been repeatedly measured with our in-house ELISA with results in the range of 310 ± 40 ng/ml [11]. Additionally, MRP8/14 levels were also measured using the commercially available Bühlmann MRP8/14 Calprotectin ELISA (Bühlmann Laboratories Schönenbuch, Zwitserland) to investigate inter-assay variation. The readers of the assay were blinded to diagnosis and disease activity. Treating physicians were blinded to the MRP8/14 serum levels.

### Statistical analysis

Patients starting etanercept and adalimumab were analyzed together, as the number of patients treated with adalimumab was too small to analyze separately. Although the two drugs are different in structure, both drugs are TNF-alpha blockers, and we therefore expect the effect on MRP8/14 levels in responders/non-responders to be comparable. Descriptive statistics are presented as absolute frequencies, as median values and IQR, or as mean and SD, as appropriate. The chi-square test was used to compare categorical characteristics of responders with those of non-responders. The Mann–Whitney *U* test was used for comparison of continuous variables. Correlations between serum level of MRP8/14 and clinical variables were assessed using Spearman’s correlation coefficient. Correlation between the in-house ELISA and the Bühlmann ELISA was assessed using Pearson’s correlation coefficient. The Wilcoxon signed rank test was used to analyze differences in MRP8/14 levels at paired time points. Receiver operator characteristic (ROC) analyses were performed to determine optimal cutoff levels separately for both assays to predict response to treatment and flare within 6 months after treatment discontinuation. Cutoff values were determined using the Youden index [20].

Univariate logistic regression models were fitted to assess the association between treatment response and baseline MRP8/14 levels. Linear regression was used to evaluate the relationship between change in JADAS-10 disease activity and baseline MRP8/14 serum levels. Change in JADAS-10 was defined as the difference between baseline and follow-up JADAS-10. Multivariable linear models were fitted for change in JADAS-10 to correct for other possible predictor variables and to assess the additional value of MRP8/14 in predicting clinical response. These variables were specified beforehand based on preexisting knowledge of their relationship with serum levels of MRP8/14 and/or the response to treatment (age at onset of JIA, baseline JADAS-10, number of previously used DMARDs, gender, baseline CHAQ score, ESR, disease duration) [4, 5, 21]. Missing data were handled using the chained equations multiple imputation command *ice* in Stata. Ten imputed datasets were created. Patients on adalimumab or etanercept were compared, and were imputed together due to having identical characteristics. Of the baseline JIA core set variable data (including VAS pain, evaluated by patient/parent) 3.6 % were missing (median of 0 missing values per patient (range 0–3). At the last available follow up within 6 months of treatment 9.3 % of the JIA core set variable data were missing (including VAS pain, median of 0 missing values per patient (range 0–7)). Analyses were performed with IBM SPSS Statistics for Windows Version 21.0, Stata/SE version 13.0 and Prism (v5, GraphPad Software, La Jolla California USA).

## Results

### Baseline characteristics

Baseline serum samples were available from 88 patients with non-systemic JIA to measure MRP8/14 levels with the in-house ELISA and of these 81 were available to perform both in-house and commercial ELISA. Characteristics of both the patients who started TNF inhibitors and those who discontinued etanercept are summarized in an additional table (see Additional file 1). Median MRP8/14 (ng/ml) in patients who started TNF-inhibiting treatment was 1,289 (IQR 795–2809). MRP8/14 serum levels were significantly correlated to ESR at baseline (Spearman’s rho 0.440, *p* <0.001). Presence of rheumatoid factor, CHAQ at baseline, number of active joints and disease activity expressed as JADAS-10 were not correlated with MRP8/14 levels.

### Clinical response to treatment

A total of 25 % of patients (n = 22) did not achieve an ACRpedi50 response or higher and were therefore considered non-responders to treatment. The remaining 66 patients were responders. Of these 66 patients, 46 achieved ACRpedi70, and 31 patients reached a state of inactive disease. Mean JADAS-10 score at the last available follow up within 6 months (median 3.2 months (IQR 2.6 − 5.0)) was 5.7 (±5.4).

Baseline characteristics of responders and non-responders are shown in Table 1. No significant differences were found between responders and non-responders. Data on the use of concomitant medication can be found in Additional file 1, Table S1A. There were no significant differences between responders and non-responders in concomitant medication such as systemic steroids (32 % of responders vs 18 % of non-responders, *p* = 0.388) or DMARDs (91 % of responders vs 73 % of non-responders, *p* = 0.064).

**Table 1:** Differences in baseline characteristics between responders and non-responders

Baseline characteristic	Responders	Non-responders
	(n = 66)	(n = 22)
Female gender, n (%)	48 (73)	18 (82)
Age at onset of JIA in years, median (IQR)	10.0 (4.2–12.3)	9.4 (3.5–13.7)
Disease duration in years, median (IQR)	2.4 (1.1–4.9)	2.3 (0.8–7.7)
JADAS-10, median (IQR)	20 (14–21)	17 (11–22)
CHAQ score, median (IQR)	1.5 (0.7–2.2)	1.3 (0.6-2.0)
Number of active joints, median (IQR)	11 (5–18)	8 (2–16)
ESR in mm/h, median (IQR)	16 (9–28)	12 (7–18)
Number of previously used DMARDs, median (IQR)	1 (1–2)	1 (1–2)

*JIA* juvenile idiopathic arthritis, *JADAS* juvenile arthritis disease activity score, *IQR* interquartile range, *ESR* erythrocyte sedimentation rate, *CHAQ* child health assessment questionnaire, *DMARDs* disease modifying anti-rheumatic drugs

### MRP8/14 serum levels and response to treatment

**Fig. 1:**
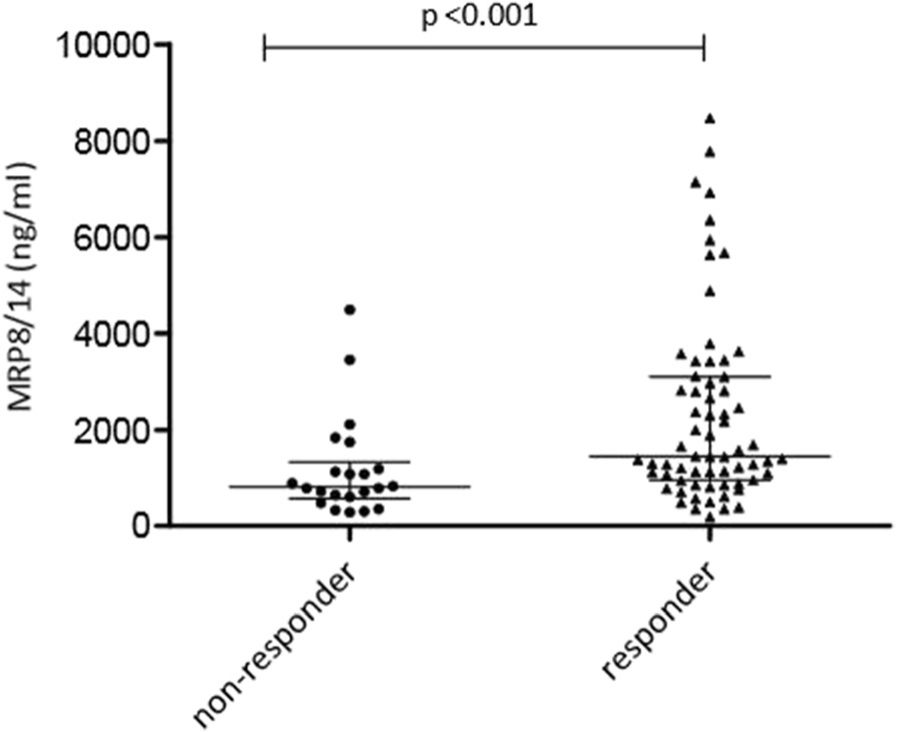
Differences in myeloid related protein (*MRP*)8/14 serum levels between non-responders and responders before starting treatment

Baseline MRP8/14 serum levels were higher in responders (median MRP8/14 of 1466 ng/ml (IQR 1045–3170)) compared to non-responders (median MRP8/14 of 812 ng/ml (IQR 570–1178), p < 0.001) (Fig. 1). If we only compare patients who achieved inactive disease with those not achieving inactive disease, these differences are also present: inactive disease vs no inactive disease: median MRP 8/14 at start (ng/ml) 2287 (IQR 1053–3672) vs 1174 (IQR 704–2030), p = 0.005.

On univariate logistic regression this resulted in an odds ratio of 1.5 (95 % CI 1.1, 2.1) for achieving at least an ACRpedi 50 response per 500 units of MRP (ng/ml). Baseline MRP8/14 serum levels were significantly associated with change in JADAS-10 on linear regression analysis (β = 0.636 per 500 unit increase in ng/ml, 95 % CI 0.254, 1.018, *p* = 0.001).

### Use of MRP8/14 as a prognostic marker for response to treatment

Based on receiver operator characteristic (ROC) curve analysis, a cutoff value was identified for prediction of response to treatment (ACR pedi50 or higher). Sensitivity, specificity and likelihood ratios are given in Table 2.

**Table 2:** Sensitivity, specificity and likelihood ratios for the determined cutoff value of myeloid related protein (MRP)8/14 to predict response to anti-TNF treatment

Accuracy measure	
Cutoff level MRP8/14 (ng/ml)	1,193
Sensitivity	66 %
Specificity	81 %
Positive likelihood ratio	3.4
Negative likelihood ratio	0.4
Youden index	0.47
Area under the curve	0.76

### Added value of MRP8/14 in prediction of response

We constructed a multivariable linear regression model including known predictors (age at onset of JIA, baseline JADAS-10, number of previously used DMARDs, gender, baseline CHAQ score, ESR, disease duration) and subsequently added MRP8/14 to the model. In this multivariable model MRP8/14 was still significantly associated with change in JADAS-10 (corrected β = 0.472 per 500 units increase in ng/ml, 95 % CI 0.161, 0.782, *p* <0.001). The only other variable significantly associated with change in JADAS-10 was baseline JADAS-10 (corrected β = 0.678, 95 % CI 0.434, 0.921, *p* <0.001). The variables in the model without MRP8/14 serum levels explained 50 % of the variance in change in JADAS-10 within 6 months of treatment (*R*^2^ = 0.50). Adding MRP8/14 to this model resulted in a slightly better predictive model, with an *R*^2^ value of 0.54 (*p* = 0.004).

### Change in MRP8/14 levels after treatment

**Fig. 2:**
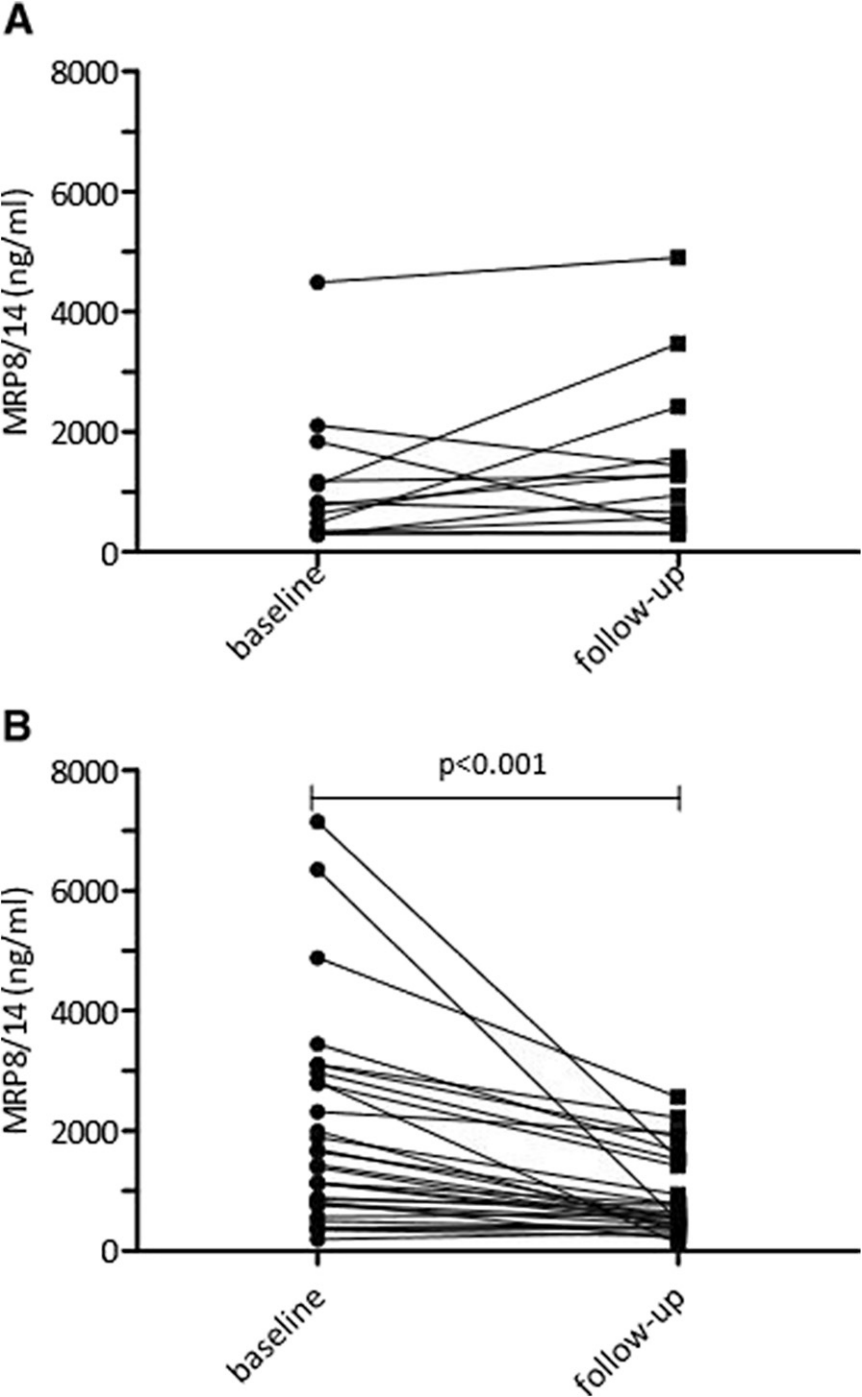
**a** Change in myeloid related protein (*MRP*)8/14 serum levels in non-responders pre-treatment compared to levels after treatment for an average of 3.2 months. **b** Change in MRP8/14 serum levels in responders pre-treatment compared to levels after treatment for an average of 3.2 months

A follow-up measurement within 5 months after starting treatment was available for 43 patients; 14 of these were categorized as being non-responders. Treatment with TNF inhibitors significantly lowered MRP8/14 serum levels in responders (*p* <0.001) (Fig. 2b), but not in non-responders (Fig. 2a). Patients who achieved inactive disease had a greater change in MRP than those who did not achieve inactive disease (median change in MRP 8/14 (ng/ml) -1034 (IQR -2723 to -401) vs -486 (IQR -1084 to 209)). Change in MRP was significantly correlated with change in JADAS10 (Spearman’s rho: 0.421, *p* = 0.006). There were no significant differences in change in MRP levels after anti-TNF treatment between patients who were or were not treated concomitantly with systemic steroids and/or DMARDs.

### Association of MRP8/14 level and flare after etanercept withdrawal after successful treatment

**Fig. 3:**
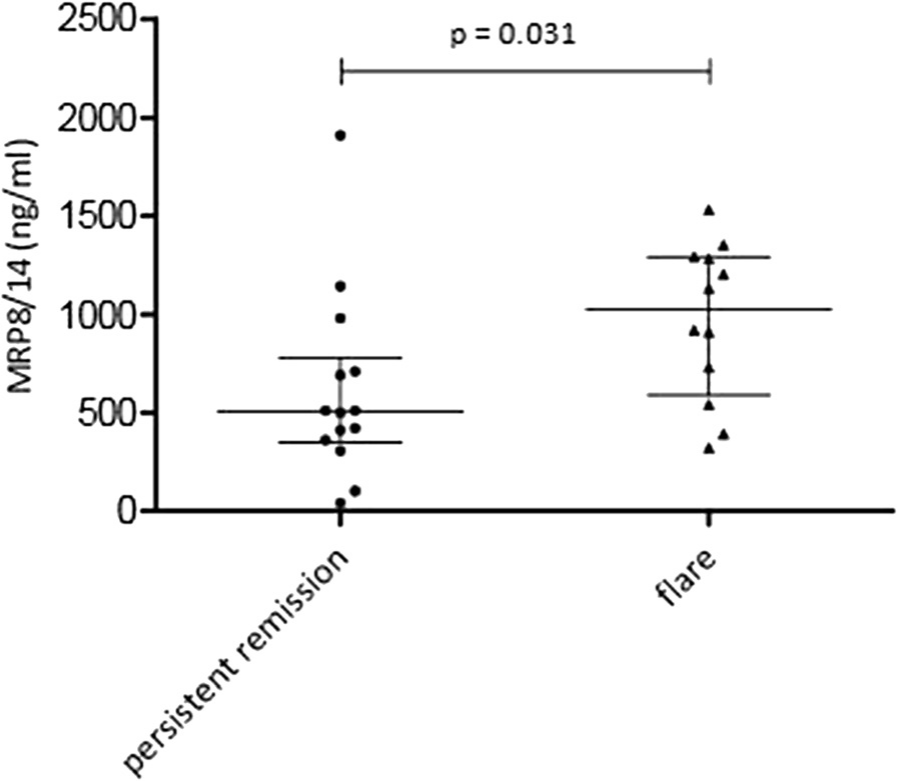
Differences in myeloid related protein (*MRP*)8/14 at time of medication discontinuation between patients who then had persistent remission and patients who had flares

Patients who had flares within 6 months (n = 12) after discontinuation of etanercept had higher MRP levels at discontinuation than patients who did not have flares (n = 14) (*p* = 0.031, median 1,025 ng/ml (IQR 588–1288) vs 505 ng/ml (IQR 346–778) (Fig. 3)). Patients discontinuing medication were allowed to have a PGA between 0 and 10 mm. Of the 26 patients discontinuing treatment, 14 patients had a PGA >0 (median PGA of 3). MRP levels were comparable between the patients who had a PGA of 0 and patients who had a PGA >0 at discontinuation of treatment (*p* = 0.432). Full disease activity parameters for all patients at discontinuation can be found in Additional file 1, Table S2.

The cutoff value for prediction of a flare after etanercept withdrawal and the prognostic accuracy are reported in Table 3.

**Table 3:** Sensitivity, specificity and likelihood ratios for the determined cutoff value of myeloid related protein (MRP)8/14 predicting a flare within 6 months

Accuracy measure	
Cutoff level MRP8/14 (ng/ml)	720
Sensitivity	75 %
Specificity	79 %
Positive likelihood ratio	3.5
Negative likelihood ratio	0.5
Youden index	0.54
Area under the curve (95 % CI)	0.75 (0.55, 0.95)

### Validation for routine use by commercial ELISA

Commercial MRP8/14 ELISA is available but is not validated for use as a monitoring tool for anti-TNF therapy. Therefore, we aimed to validate our findings with the commercial Bühlmann MRP8/14 Calprotectin ELISA to make MRP8/14 as a marker for anti-TNF therapy widely available. Measurements using the in-house ELISA correlated very well with those from the Bühlmann ELISA (Pearson’s rho 0.902, *p* <0.001). Although MRP8/14 levels appeared to be 3-fold to 4-fold higher when they were measured with the Bühlmann ELISA, the associations between MRP8/14 and response, on both the achievement of ACRpedi 50 or higher and on change in JADAS-10 were comparable. For predicting response to anti-TNF treatment and flares after discontinuation in clinical remission, the in-house ELISA and the Bühlmann ELISA had the same accuracy. Full results for the Bühlmann ELISA data are shown in Additional file 2.

## Discussion

In this study we show that MRP8/14 can predict response to anti-TNF treatment, although it has little additive value to other clinical factors. Patients who responded to anti-TNF treatment had higher levels of MRP8/14 at the start of that treatment than patients who did not respond. Disease activity declined more in patients with higher levels of MRP8/14. In responders, these levels decreased after initiation of treatment, and in non-responders MRP8/14 levels were constant. When the disease had become inactive and treatment with etanercept could be stopped, MRP8/14 levels appeared to be higher for patients in whom the disease flared than in patients who did not experience a flare. The prognostic accuracies of the in-house ELISA and the Bühlmann ELISA were comparable, although the cutoff levels were different.

In recent years, MRP8/14 has been widely studied as potential predictor of disease activity and response to treatment in rheumatic and other inflammatory diseases. In rheumatoid arthritis (RA), MRP8/14 has been used as a predictor for response to biologic treatment [22]. In JIA in particular, MRP8/14 levels are highly predictive of disease activity and of disease flares in systemic JIA [11]. Also in enthesitis-related arthritis there is a relationship between MRP8/14 levels and disease activity [23]. In a more heterogeneous group of JIA patients, MRP8/14 levels have been shown to predict response to MTX treatment [12]. The univariate odds ratio for achieving an ACRpedi50 response or higher following MTX treatment [12] is comparable to the odds ratio found in the present study following anti-TNF treatment. Using the same ELISA to measure MRP8/14 levels, the average serum levels of MRP8/14 in that study were higher than in our patients. Patients in the present study mostly had MRP8/14 levels comparable to patients who did not respond to MTX treatment [12]. This is not surprising as failure of MTX treatment is an eligibility criterion for treatment with biologic agents. Therefore there may be a possibility of using MRP8/14 levels to decide which patient is more likely to respond to MTX and which patient may be better off with biologic treatment right away. Unfortunately we did not have MRP8/14 serum levels for our patients at the start of MTX treatment.

There are well-established experimental ELISA protocols for MRP8/14, however, these are not available for use in routine laboratories. The commercially available Bühlmann ELISA kit has already been demonstrated to perform well in analyzing patient serum samples. To obtain reliable results in the range of MRP8/14 concentrations found at different levels of disease activity in JIA, serial dilution of individual sera to obtain reliable results is necessary [24]. In addition, the level of MRP8/14 concentrations analyzed with the two assays varied substantially. Therefore, a direct comparison of results obtained with one ELISA with a result from a different assay should not be made. Both methods were equally accurate in predicting response to treatment and flares after discontinuation in clinical remission.

For a biomarker to be used in informing therapeutic choice, it will have to fulfill certain requirements. It has to able to predict a certain outcome and the predictive value has to be validated. It has to have additional value on top of other known predictors. Additionally the prediction should have therapeutic consequences. MRP8/14 has been shown to be associated with response to treatment both in this and previous studies in JIA on both relative (ACRpedi50) and absolute measures (change in JADAS-10). Here, MRP8/14 added to the prediction of change in disease activity, though its additive value was small. Some of the responders and non-responders to treatment had comparable MRP8/14 serum levels, and sensitivity and specificity were not optimal for any cutoff value, which is in line with the study by Moncrieffe et al. [12]. Because prediction is not perfect, therapeutic decisions cannot only be based on MRP8/14 levels. However, it is unlikely that a single biomarker will ever be able to perfectly predict response in the heterogeneous pool of JIA patients. This biomarker has the advantage that it is a relatively stable protein and easily measurable in serum, in contrast to, for instance, cytokines such as TNF or IL-1beta. Therefore, MRP8/14 could play a supporting role in response prediction models for response to treatment including clinical as well as laboratory measures, which are under investigation for both JIA and RA [4, 25–29]. More importantly, MRP8/14 might be used to objectively monitor disease activity as it reflects treatment response and confirms disease activity independently from clinical parameters, and might be useful as an early marker of response in clinical trials. In the future, additional parameters are needed that could contribute to a diagnostic panel to guide treatment decisions. MRP expression could enable identification of further biomarkers by defining and dissecting treatment response groups.

For prediction of flares after discontinuation of treatment, MRP8/14 can possibly be used as a prediction tool. MRP8/14 serum levels have already been shown to be predictive of flares after the discontinuation of MTX in JIA patients [10]. We show that this is true to the same extent after stopping etanercept in patients with non-systemic JIA after inactive disease has been achieved. Additionally, we found cutoff values comparable to the earlier specified cutoff value for the in-house ELISA [10, 24]. Still, the cutoff values did not perfectly predict flare or persistent remission after discontinuation. For clinical practice this means that we have to keep searching for additional features that will provide a better prediction model.

## Conclusions

In conclusion, serum levels of MRP8/14 are associated with response to treatment with etanercept in patients with non-systemic JIA. MRP8/14 serum levels decrease together with disease activity in responders to etanercept, however, it is of minor predictive value when added to a prediction model with known predictors. MRP8/14 serum levels may be useful in practice to predict flares in patients in clinical remission after cessation of etanercept. They can also be determined using a commercially available ELISA kit.

## Additional files

Additional file 1: Table S1. Characteristics of patients starting anti-TNF treatment who were included in the study (DOCX 21 kb) Additional file 2:
**Results of Bühlmann ELISA.** Results of repeat analysis of the same samples using a commercially available ELISA kit (DOCX 88 kb)

## Abbreviations

ABC: Arthritis and Biologicals in Children
ACR: American College of Rheumatology
BIKER: German Registry for Biologics in Pediatric Rheumatology
CHAQ: childhood health assessment questionnaire
CHARMS: Childhood arthritis response to medication study
DMARD: disease-modifying anti-rheumatic drug
ELISA: enzyme-linked immunosorbent assay
ESR: erythrocyte sedimentation rate
IL: interleukin
IQR: interquartile range
JADAS: juvenile arthritis disease activity score
JIA: juvenile idiopathic arthritis
MRP: myeloid related protein
MTX: methotrexate
RA: rheumatoid arthritis
ROC: receiver operator characteristic
TNF: tumor necrosis factor
VAS: visual analog scale
