# Supplementary material for: MRP8/14 serum levels as a predictor of response to starting and stopping anti-TNF treatment in juvenile idiopathic arthritis
Source: Arthritis Res Ther. 2015 Aug 7;17(1):200. doi: 10.1186/s13075-015-0723-1 (PMC4528380; doi:10.1186/s13075-015-0723-1)
Supplement: Additional file 1: Table S1. — Characteristics of patients starting anti-TNF treatment who were included in the study (DOCX 21 kb) [file 13075_2015_723_MOESM1_ESM.docx]

**Supplemental table S1A Characteristics of patients starting anti-TNF treatment**

| **Baseline characteristics** | **N=88** |
| --- | --- |
| Female, n (%) | 66 (75) |
| Age at onset JIA in years, median (IQR) | 10.0 (3.9-12.3) |
| Age at start first biological in years, median (IQR) | 12.8 (9.9-15.6) |
| JIA disease duration before start biological in years, median (IQR) | 2.3 (0.9-6.0) |
| ANA positive, n/N (%) | 25/76 (33) |
| RF positive, n/N (%) | 13/80 (16) |
| Category JIA, n (%) |  |
| Polyarticular RF negative | 33 (38) |
| Polyarticular RF positive | 13 (15) |
| Oligoarticular extended | 24 (27) |
| Oligoarticular persistent | 5 (6) |
| Psoriatic arthritis | 9 (10) |
| Enthesitis related arthritis | 4 (5) |
| Previously used medications, n (%) |  |
| Systemic prednisone | 42 (48) |
| MTX | 85 (97) |
| DMARD other than MTX | 26 (30) |
| Biological started, n (%) |  |
| Etanercept | 81 (92) |
| Adalimumab | 7 (8) |
| Concomitant co-medication at start biological, n (%) |  |
| Intra-articular steroids | 3 (3) |
| Systemic prednisone | 25 (28) |
| MTX | 74 (84) |
| DMARD other than MTX | 3 (3) |
| Disease activity parameters at baseline, median (IQR) |  |
| VAS physician (0-100) | 54 (30-68) |
| CHAQ total (0-3) | 1.50 (0.75-2.1) |
| VAS pain (0-100) | 56 (25-72) |
| VAS wellbeing (0-100) | 53 (25-70) |
| Active joints | 10 (5-17) |
| Limited joints | 6 (2-14) |
| ESR | 13 (8-27) |
| JADAS-10 (0-40), mean (SD) | 18 (7) |
| MRP8/14 measured by in-house ELISA (ng/ml), median (IQR) | 1289 (795-2809) |
| MRP8/14 measured by Bühlmann ELISA (ng/ml), median (IQR) | 4763 (2795-8701) |

JIA= juvenile idiopathic arthritis, IQR= interquartile range, ANA= anti-nuclear antibodies, RF= rheumatoid factor, DMARD= disease modifying anti-rheumatic drug, MTX= methotrexate, VAS= visual analogue scale, CHAQ = childhood health assessment questionnaire, ESR= erythrocyte sedimentation rate, JADAS= juvenile arthritis disease activity score

**Supplemental table S1B Characteristics of patients discontinuing etanercept in clinical remission**

| **Baseline characteristics** | **Flare (n=12)** | **Persistent remission (n=14)** |
| --- | --- | --- |
| Female, n (%) | 9 (75) | 9 (64) |
| Category JIA, n (%) |  |  |
| Polyarticular RF negative | 6 (50) | 8 (57) |
| Polyarticular RF positive | - | 1 (7) |
| Oligoarticular extended | - | 4 (29) |
| Oligoarticular persistent | 3 (25) | - |
| Psoriatic arthritis | 1 (8) | - |
| Enthesitis related arthritis | 2 (17) | 1 (7) |
| MTX treatment at time of discontinuation, n (%) | 5 (42) | 4 (28) |
| MRP8/14 measured by in-house ELISA (ng/ml), median (IQR) | 1025 (588-1288) | 505 (346-778) |
| MRP8/14 measured by Bühlmann ELISA (ng/ml), median (IQR) | 3835 (2146-4806) | 1415 (1099-2863) |

JIA= juvenile idiopathic arthritis, IQR= interquartile range, RF= rheumatoid factor, MTX= methotrexate,

**Supplemental table S2 Disease activity at discontinuation of treatment**

| **Baseline variable** | **Patients who had continuous remission** | **Patients who had a flare** | **p-value (Mann Whitney U)** |
| --- | --- | --- | --- |
| PGA, median (IQR) | 0 (0-4) | 2 (0-3) | 0.432 |
| CHAQ score, median (IQR) | 0.13 (0.00-0.38) | 0.00 (0.00-0.19) | 0.247 |
| VAS pain, median (IQR) | 0 (0-4) | 1 (0-5) | 0.894 |
| VAS wellbeing, median (IQR) | 0 (0-3) | 2 (0-3) | 0.894 |
| Active joints, median (IQR) | 0 (0-0) | 0 (0-0) | 1.000 |
| Limited joints, median (IQR) | 0 (0-0) | 0 (0-0) | 0.940 |
| ESR, median (IQR) | 12 (6-17) | 9 (4-14) | 0.432 |

PGA= Physician Global Assessment (range 0-100), IQR= interquartile range, VAS= visual analogue scale (range 0-100), CHAQ = childhood health assessment questionnaire (range 0-3), ESR= erythrocyte sedimentation rate
