# Supplementary material for: MRP8/14 serum levels as a predictor of response to starting and stopping anti-TNF treatment in juvenile idiopathic arthritis
Source: Arthritis Res Ther. 2015 Aug 7;17(1):200. doi: 10.1186/s13075-015-0723-1 (PMC4528380; doi:10.1186/s13075-015-0723-1)
Supplement: Additional file 2: — Results of Bühlmann ELISA. Results of repeat analysis of the same samples using a commercially available ELISA kit (DOCX 88 kb) [file 13075_2015_723_MOESM2_ESM.docx]

**Supplementary file: results of Bühlmann ELISA**

*MRP8/14 levels at baseline and response to treatment*

MRP8/14 serum levels were significantly correlated to ESR at baseline (Spearman’s rho 0.361, p=0.001 (Bühlmann ELISA)).

Baseline MRP8/14 serum levels were higher in responders (median in responders was 5556 (IQR 3092-10008)) compared to non-responders (median 2504 (IQR 1292-3950), p<0.001)) (Supplementary Figure S1).

In a univariate logistic regression this resulted in an OR of 1.2 (95% CI: 1.0-1.3) for achieving at least an ACRpedi 50 response per 500 units of MRP (ng/ml) for the Bühlmann ELISA measurements.

*Prediction of response corrected for other variables*

Baseline MRP8/14 serum levels were significantly associated with change in JADAS-10 in a univariate linear regression analysis (β = 0.245, 95% CI 0.116 to 0.375, p<0.001 for the Bühlmann ELISA). IN the corrected multivariable analysis the corrected β was 0.197 per 500 units increase in ng/ml, 95% CI 0.087 to 0.306, p<0.001. The change in explained variance was identical: 4%.

*Use of MRP8/14 as a prognostic marker for response to treatment*

The in-house ELISA and the Bühlmann ELISA had the same accuracy for predicting response to anti-TNF treatment, the accuracy of the Bühlmann ELISA is shown in Table S3.

**Table S3 Sensitivity, specificity and likelihood ratios for the determined cut-off of MRP8/14 predicting response to anti-TNF treatment, Bühlmann ELISA**

|  | **Bühlmann ELISA** |
| --- | --- |
| Cut-off level MRP8/14 (ng/ml) | 4387 |
| Sensitivity | 67% |
| Specificity | 81% |
| Positive likelihood ratio | 3.4 |
| Negative likelihood ratio | 0.4 |
| Youden index | 0.47 |
| AUC | 0.77 |

AUC= area under the curve

*Change in MRP8/14 levels after treatment*

Of 34 patients, enough serum was available to determine MRP in the follow-up sample. Of these patients 11 could be categorized as non-responders. Treatment with etanercept lowered MRP8/14 serum levels significantly only in responders (p<0.001 for both ELISAs) (Supplementary Figure S2B), but not in non-responders (Supplementary Figure S2A)). Change in MRP was significantly correlated to change in JADAS10 (Spearman’s rho: 0.581, p=0.001 (Bühlmann ELISA)).

*Association of MRP8/14 level and flare after etanercept withdrawal after successful treatment*

Patients who flared within 6 months (n=12) after the discontinuation of etanercept had higher MRP levels at discontinuation than patients who did not flare (n=14) (p= 0.013, median 3835 (IQR 2146-4806) vs. 1415 (IQR 1099-863) (Bühlmann ELISA), Supplementary Figure S3).

Cut-off for the prediction of a flare after etanercept withdrawal plus their prognostic accuracy are given in Table S4.

**Table S4 Sensitivity, specificity and likelihood ratios for various cut-off values of MRP8/14 predicting a flare within 6 months**

| **Accuracy measure** | **Bühlmann ELISA** |
| --- | --- |
| Cut-off level MRP8/14 (ng/ml) | 2045 |
| Sensitivity | 83% |
| Specificity | 71% |
| Positive likelihood ratio | 2.9 |
| Negative likelihood ratio | 0.2 |
| Youden index | 0.55 |
| AUC (95% CI) | 0.79 (0.61 to 0.96) |

AUC= area under the curve
